# Electrical Stimulation Influences Satellite Cell Proliferation and Apoptosis in Unloading-Induced Muscle Atrophy in Mice

**DOI:** 10.1371/journal.pone.0030348

**Published:** 2012-01-12

**Authors:** Bao-Sheng Guo, Kwok-Kuen Cheung, Simon S. Yeung, Bao-Ting Zhang, Ella W. Yeung

**Affiliations:** Muscle Physiology Laboratory, Department of Rehabilitation Sciences, Hong Kong Polytechnic University, Hung Hom, Kowloon, Hong Kong; McMaster University, Canada

## Abstract

Muscle atrophy caused by disuse is accompanied by adverse physiological and functional consequences. Satellite cells are the primary source of skeletal muscle regeneration. Satellite cell dysfunction, as a result of impaired proliferative potential and/or increased apoptosis, is thought to be one of the causes contributing to the decreased muscle regeneration capacity in atrophy. We have previously shown that electrical stimulation improved satellite cell dysfunction. Here we test whether electrical stimulation can also enhance satellite cell proliferative potential as well as suppress apoptotic cell death in disuse-induced muscle atrophy. Eight-week-old male BALB/c mice were subjected to a 14-day hindlimb unloading procedure. During that period, one limb (HU-ES) received electrical stimulation (frequency: 20 Hz; duration: 3 h, twice daily) while the contralateral limb served as control (HU). Immunohistochemistry and western blotting techniques were used to characterize specific proteins in cell proliferation and apoptosis. The HU-ES soleus muscles showed significant improvement in muscle mass, cross-sectional area, and peak tetanic force relative to the HU limb (p<0.05). The satellite cell proliferative activity as detected within the BrdU^+^/Pax7^+^ population was significantly higher (p<0.05). The apoptotic myonuclei (detected by terminal deoxynucleotidyl transferase-mediated dUTP nick end labeling) and the apoptotic satellite cells (detected by cleaved Poly [ADP-ribose] polymerase co-labeled with Pax7) were reduced (p<0.05) in the HU-ES limb. Furthermore the apoptosis-inducing factor and cleaved caspase-3 were down-regulated while the anti-apoptotic Bcl-2 protein was up-regulated (p<0.05), in the HU-ES limb. These findings suggest that the electrical stimulation paradigm provides an effective stimulus to rescue the loss of myonuclei and satellite cells in disuse muscle atrophy, thus maintaining a viable satellite cell pool for subsequent muscle regeneration. Optimization of stimulation parameters may enhance the outcome of the intervention.

## Introduction

Skeletal muscles are highly adaptive for functional changes and external insults. Adult skeletal muscle fibers are terminally differentiated tissues, such that regeneration of skeletal muscle from injury depends on recruitment of resident satellite cells. Upon appropriate stimulatory signals, satellite cells will activate from the quiescent stage, undergo active proliferation and myogenic differentiation, and subsequently fuse with pre-existing fibers or fuse with other myoblasts forming myotubes which mature into new myofibers (hyperplasia) [1], [2]. Therefore, the survival and proliferative potential of satellite cells determines the regenerative capacity of skeletal muscles (for review, see [3], [4]). Deficiency in the regenerative capacity of skeletal muscles subjected to myotrauma results in muscle atrophy [5].

Skeletal muscle atrophy, regardless of events such as mechanical unloading after spaceflight, immobilization, bed rest, or denervation, is characterized by a decrease in protein content, fiber diameter, and force production. Mechanical unloading has been shown to reduce the numbers of satellite cells, probably due to impaired satellite cell proliferation, resulting in decreased muscle mass and protein content [6], [7], [8]. In addition to the reduced numbers of satellite cells, satellite cells isolated from the unloaded muscles failed to proliferate and differentiate into multinucleated myotubes [9]. These results further confirmed that mechanical unloading impaired both the numbers as well as the regenerative capacity of satellite cells. On the other hand, apoptosis was shown to be involved in chronically denervated muscle [5] and mechanically unloaded muscle [10]. Apart from the loss of existing myonuclei in apoptosis, satellite cells have also been shown to be susceptible to apoptosis [11], [12], [13]. As early as 48 h after mechanical unloading, BrdU-labeled satellite cells appeared to be more prone to apoptosis [11]. The principle of the nuclear domain hypothesis suggests that each defined volume of cellular territory within each myofiber is controlled by a myonucleus. Any addition or removal of myonuclei should theoretically be accompanied by an increase or decrease in myofiber size, and in turn, lead to hypertrophy or atrophy, respectively [5]. Any means, whether physical, chemical, or biological, that enhances the satellite cell proliferation and/or inhibition of apoptosis (myonuclei or satellite cells), should be efficient in rescuing skeletal muscle atrophy.

Electrical stimulation has been explored as a means of counteracting skeletal muscle atrophy in various clinical conditions, such as spinal cord injury [14], aging [15], spaceflight [16], and disuse [17], [18]. The effect of electrical stimulation depends on the atrophy model and the parameters of electrical stimulation, particularly the frequency and duration of stimulation. Low-frequency stimulation (2–20 Hz) that matches the motor unit firing pattern of a slow-twitch muscle, such as the soleus muscle, has been shown to be effective in attenuating disuse muscle atrophy [17], although the underlying mechanisms are far from clear. We have previously demonstrated that, during hindlimb suspension, application of low-frequency electrical stimulation at 20 Hz on the soleus muscles with defined timing and pulse parameters partially rescued the loss of satellite cells and improved fiber cross-sectional areas [19]. Furthermore, we have also shown that the reduced myonuclear domain caused by hindlimb unloading significantly recovered after electrical stimulation [19]. We hypothesize that the recovery of the myonuclear domain observed is a result of increase in satellite cell proliferative potential and the inhibition of cell apoptosis. Therefore, the aim of this study was to examine the effect of electrical stimulation on the proliferation of satellite cells, and apoptosis of both myonuclei and satellite cells in disuse muscle atrophy. The contractile properties of the muscle in relation to peak tetanic force (P_o_), length-tension, and force-frequency relationships were also assessed.

## Materials and Methods

### Ethics Statement

All animal handling procedures and experimental protocols were approved by The Hong Kong Polytechnic University Animal Ethics Committee (ASESC no. 07/23).

### Animals

Male BALB/c mice (8–10 wk) used in this study were housed in a temperature-controlled, 12 h light/dark cycle facility, and food and water were available *ad libitum*. The animals were acclimatized to the laboratory conditions for at least 7 days before being used in experiments.

### Experimental design

The animals were randomly assigned into control (*n* = 12) and unloaded groups (*n* = 12). The control, weight-bearing (WB) mice were allowed to move unconstrained around the cages. The hindlimb unloading procedure was achieved by tail suspension, as described previously [19], [20]. Briefly, an orthopedic adhesive tape was applied along the proximal one-third of the tail and placed through a metal ring, which attached to a metal bar on the top of a hindlimb suspension cage. This allowed the forelimbs to have contact with the grid bottom of the cage such that the animals could move and access food and water freely. The suspension height was adjusted to maintain a suspension angle of 30° and to ensure that the hindlimbs were unable to touch any supporting surface. The animals were suspended for a total of 14 days.

During unloading, one hindlimb was subjected to electrical stimulation (HU-ES) while the contralateral unloaded limb (HU) served as control. Details of the electrical stimulation protocol have previously been described [19]. Animals were anesthetized through intraperitoneal injection of a cocktail containing ketamine (100 mg/kg) and xylazine (5 mg/kg). The hindlimb to be stimulated was carefully shaved without any damage to the skin. Two electrodes were placed on the skin overlying the mid-calf region (plantarflexors) and the lateral side of the tibia (dorsiflexors). A 20-Hz frequency stimulation with a square-wave configuration and pulse width of 200 µs (CEFAR Digital Unit, Sweden) was given twice a day for 3 h (with a 2-h rest in between), for a total of 6 h of treatment per day.

### Force measurement

After 14 days of hindlimb unloading, the animal was sacrificed via cervical dislocation and the soleus muscles were isolated. The muscles were weighed and mounted to an experimental chamber with Krebs' solution (22–24°C) bubbled with 95% O_2_/5% CO_2_ (pH 7.4). The Krebs' solution had the following composition (in mM): NaCl 136, KCl 5, CaCl_2_ 1.8, MgCl_2_ 0.5, NaHCO_3_ 11.9, and NaH_2_PO_4_ 0.4. Force-length relation, force-frequency relation, and P_o_ were established. The experimental setup and protocol has been previously described in detail [21], [22]. Briefly, the muscle was mounted between a force transducer (BG-10, Kulite Semiconductor Products Inc., USA) and the arm of a motor (300B-LC, Aurora Scientific, Canada). The motor allowed known length changes to be imposed on the muscle. The force-length curve and P_o_ were established with step changes in length. All tetanic contractions were 400 ms in duration at a stimulation frequency of 100 Hz. The force-frequency relation was established by stimulating the muscle at frequencies of 10, 20, 40, 60, 80, and 100 Hz.

### Histology

Isolated soleus muscles were frozen in liquid nitrogen-chilled isopentane and cryoembedded with OCT (Tissue-Tek, Germany) for sectioning. Cross-sections of 6-µm thickness were cut from the mid-belly of each muscle. The sections were fixed in 4% paraformaldehyde in PBS (pH 7.4), rinsed, and stained using hematoxylin and eosin. Sections were examined with a light microscope (20× objective magnification) and images were captured using a Spot digital camera (Carl Zeiss MicroImaging, Inc., Germany). Images of a muscle section were captured to provide a single digital image of the entire muscle cross-sectional area. Measures of the muscle fiber cross-sectional area (in µm^2^) were obtained using Image J imaging software (National Institutes of Health, USA).

### In situ terminal deoxynucleotidyl transferase-mediated dUTP nick end labeling (TUNEL) staining

Visualization of DNA fragmentation, a marker of apoptosis, was performed by the TUNEL method using the fluorometric *in situ* cell death detection kit (Roche Applied Science, USA) according to the manufacturer's instructions. Briefly, 6-µm thick frozen cross-sections from individual muscle were fixed in 4% paraformaldehyde in PBS (pH 7.4) at room temperature for 20 min, and incubated with fluorescein-conjugated TdT enzyme at 37°C for 1 hr in the dark. Sections were incubated with rabbit anti-mouse dystrophin polyclonal antibody (1∶100 dilution; Santa Cruz Biotechnology Inc., USA) followed by an anti-rabbit IgG Rhodamine-conjugated F(ab')2 fragment incubation (1∶200 dilution; Jackson ImmunoResearch Laboratories, Inc., USA) for sarcolemmal identification. Sections were then mounted with DAPI (4′,6-diamidino-2-phenylindole) Vectashield mounting medium (Vector Laboratories Inc., USA) for nuclei counterstaining. Cross-sections were imaged (40× objective) using a fluorescent microscope (Eclipse 80i; Nikon, Tokyo, Japan). Both TUNEL- and DAPI-positive staining in nuclei located under the basal lamina and the sarcolemma were counted. Data are reported as TUNEL index, which was calculated by counting the total number of TUNEL-positive nuclei divided by the total fiber number.

### Fluorescence immunohistochemistry

Soleus muscle sections were fixed in pre-cooled acetone at 4°C and permeabilized using 0.2% Triton X-100. Non-specific binding was blocked by 5% normal horse serum. Sections were incubated with primary antibodies targeting apoptosis-related proteins that included cleaved Poly (ADP-ribose) polymerase (PARP), cytochrome c, apoptosis-inducing factor (AIF), Bax, and Bcl-2 (Cell Signaling Technology Inc., USA), followed by Alexa Fluor-conjugated secondary antibodies (Invitrogen, USA). The washed sections were mounted with DAPI-containing mountant (Vectorshield, Vector Laboratories, Inc.) for nuclei identification. Immunofluorescence signals were captured using Spot RT digital camera (Carl Zeiss MicroImaging, Inc.).

### Bromodeoxyuridine (BrdU) incorporation and detection

To assess cell proliferation, animals were injected daily with a 10 mg/ml solution of BrdU in PBS intraperitoneally during the entire 14-day unloading period. To identify proliferated satellite cells, double labeling with satellite cell marker Pax7 was performed. Fixed sections were incubated with 2N HCl for DNA denaturation. After several washes, the sections were incubated with mouse anti-BrdU monoclonal antibody (1∶100; Sigma-Aldrich, USA) and rabbit anti-Pax7 polyclonal antibody (1∶100; catalog no. ab34360, Abcam, USA) at 4°C overnight, followed by Alexa 488-conjugated donkey anti-mouse IgG and Alexa 556-conjugated donkey anti-rabbit IgG (1∶200; Invitrogen). To confirm successful BrdU incorporation, the skin tissue was harvested and sectioned as a positive control. Pax7^+^ and BrdU^+^/Pax7^+^ cells were quantified on two sections from the mid-belly region of each muscle. The number of positive cells was counted on the entire muscle sections and expressed per 1000 muscle fibers.

### Western blotting

Soleus muscles were collected for western blotting analysis as described previously [23] with minor modifications. Briefly, the muscles were homogenized on ice in 1X RIPA buffer (Cell Signaling Technology Inc.) containing protease inhibitor cocktail (Roche Applied Biosciences) and 1 mM PMSF (Merck & Co., USA). The extracted protein after centrifuging was quantified using Bradford Assay (Quick Start, Bio-Rad Laboratories, USA). Twenty-microgram protein samples were separated by SDS-PAGE and transferred onto nitrocellulose membranes (Perkin Elmer Corporation, USA). After blocking with 5% non-fat dry milk (Bio-Rad Laboratories) in PBS containing 0.05% Tween 20, the membranes were probed with primary antibodies for cleaved PARP, cytochrome c, AIF, Bax, and Bcl-2 antibodies (1∶1000; Cell Signaling Technology Inc.), and subsequently with near-infrared dye-conjugated secondary IgG. Immunoreactive bands were detected using the Odyssey® Infrared Imaging System (LI-COR Biosciences, USA) and quantified using 1-D image analysis system (LI-COR Biosciences).

### Statistical analysis

One-way ANOVA with Bonferroni *post hoc* comparisons was used to assess differences between groups for each dependent variable. For variables that are not normally distributed (assessed by Shapiro-Wilk test), non-parametric Kruskal-Wallis statistics was used. Statistical significance was set at p<0.05. All data are presented as mean ± standard error of the mean (SEM).

## Results

### Body weight, soleus muscle mass, and cross-sectional area

Body weight did not differ between groups (WB, HU, HU-ES) at the start of the experiment or at the end of the 14-day unloading period. The soleus muscle mass was normalized to body weight to compare among groups.

The soleus muscle wet mass of the HU group was reduced by 68.8% ± 2.8% when compared with the WB group (p<0.001). Electrical stimulation (HU-ES group) produced a significant improvement of muscle mass to 82.0% ± 3.1% compared to WB controls (Table 1). In comparison with the HU group, electrical stimulation (HU-ES) partially attenuated the decline in muscle mass by approximately 80%.

**Table 1:** Body weight and muscle mass.

	Body Weight (g)		Muscle Mass (mg)	Normalized Muscle Mass (mg/g)
	Day 0	Day 14		
**WB**	23.47 ± 0.25	24.89 ± 0.24	17.92 ± 0.34	0.72 ± 0.01
**HU**	23.96 ± 0.46	23.05 ± 0.55	12.33 ± 0.49^*^	0.54 ± 0.03^*^
**HU-ES**	23.96 ± 0.46	23.05 ± 0.55	14.67 ± 0.56^*#^	0.64 ± 0.03^*#^

Body weight (BW), soleus muscle mass, and normalized muscle mass in weight-bearing (WB), hindlimb-unloaded (HU), and electrically stimulated (HU-ES) groups. *p<0.05, significant difference compared to WB group; ^#^p<0.05, significant difference compared to HU group. Values are means ± SEM.

The mean muscle fiber cross-sectional area of the soleus in the HU group was reduced by 43.8% ± 7.9% compared to that of the WB group (HU: 1036.77 ± 145.26 µm^2^; WB: 1844.94 ± 98.41 µm^2^; p<0.001) (Table 2). A significant difference was observed in the fiber cross-sectional area between the HU-ES and HU groups in which electrical stimulation partially attenuated the unloading-induced reduction in the fiber cross-sectional area (HU-ES: 1481.55 ± 121.44 µm^2^; p<0.05).

**Table 2:** Cross-sectional area and muscle force measurements.

	CSA (um^2^)	P_o_ (mN)	P_o_ normalized to CSA (mN mm^−2^)	Optimal length (mm)
**WB**	1844.94 ± 98.41	329.55 ± 9.75	544.87 ± 7.35	9.44 ± 0.08
**HU**	1036.77 ± 145.26*	170.40 ± 19.00*	342.13 ± 17.30*	8.04 ± 0.07*
**HU-ES**	1481.55 ± 121.44*^#^	252.49 ± 15.62*^#^	446.37 ± 20.57*^#^	8.83 ± 0.11*^#^

Cross sectional area (CSA), maximal tetanic force (P_o_) with or without normalization to CSA, and optimal length in soleus muscles in weight-bearing (WB), hindlimb-unloaded (HU), and electrically stimulated (HU-ES) groups. *p<0.05, significant difference compared to WB group; ^#^ p<0.05, significant difference compared to HU group. Values are means ± SEM.

To assess possible systemic effects of electrical stimulation on the contralateral hindlimb unloaded soleus muscle (i.e., HU group), control experiments were performed. We compared the effects of 14-day unloading without electrical stimulation intervention on the soleus muscles with the HU group. All parameters measured were compared and no difference was observed.

### Contractile properties of the soleus muscle

The P_o_ was normalized to the cross-sectional area (Table 2). The normalized P_o_ developed by the soleus muscle in the HU group showed a large reduction by 37.2% ± 3.2% when compared to WB control (HU: 342.13 ± 17.30 mN mm^−2^; WB: 544.87 ± 7.35 mN mm^−2^; p<0.001). Electrical stimulation significantly prevented the decrease in P_o_ (446.37 ± 20.57 mN mm^−2^; p<0.001) compared to the HU group.

**Figure 1:**
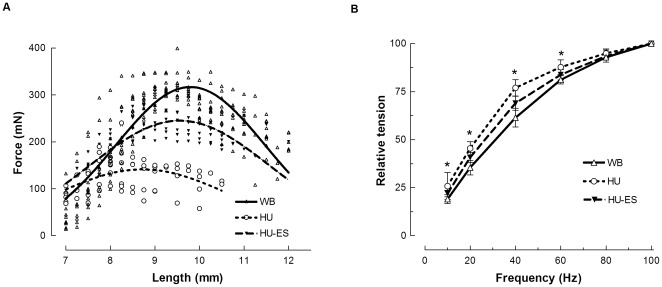
Force-length and force-frequency relation of the soleus muscle in the weight-bearing (WB), hindlimb-unloaded (HU), and electrically stimulated (HU-ES) groups. (A) Curves showing the force-length relationship obtained for one soleus muscle. (B) Force-frequency relations of the muscles normalized to 100-Hz stimulation under each condition. *p<0.05, significant difference between the WB and HU group. Values are means ± SEM.

Fig. 1*A* illustrates the length-tension relations of the soleus muscles under different conditions. As a consequence of unloading, there was a shift in the optimum length (L_o_) for force to a shorter muscle length. We observed a leftward shift of L_o_ in the HU group when compared to the WB control group (HU: 8.04 ± 0.07 mm; WB: 9.44 ± 0.08 mm; p<0.001). Electrical stimulation was able to attenuate the unloading-induced change to L_o_ (8.83 ± 0.11 mm; p<0.001).

Fig. 1*B* shows the force-frequency relations of the soleus muscles normalized to 100-Hz stimulation as 100% of control. Compared with WB control, hindlimb unloading (HU group) resulted in a relative reduction of force at low-stimulation frequencies (10, 20, 40, and 60 Hz; p<0.05). The force-frequency relation for the HU-ES group was not significantly different from that of the WB control.

### Satellite cell proliferation

**Figure 2:**
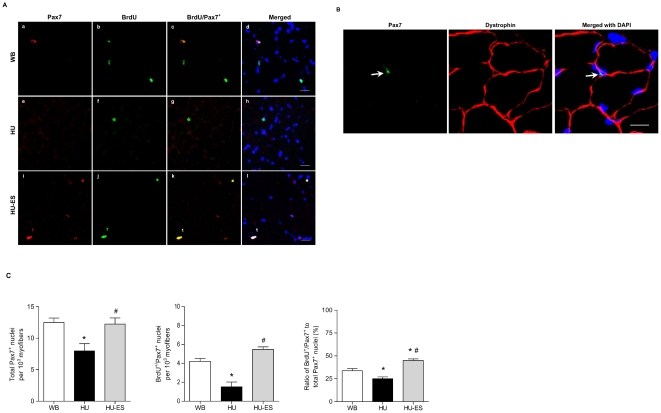
Satellite cell proliferation. (A) Immunohistochemically stained cross-sections of soleus muscle from weight-bearing (WB), hindlimb-unloaded (HU), and electrically stimulated (HU-ES) groups showing expression of Pax7 (red) and BrdU (green) incorporation counterstained with DAPI (blue) for nuclei identification. Yellow staining represents Pax7 and BrdU double-positive nuclei. Scale bar = 25 µm. (B) Co-immunostaining of Pax7 and dystrophin on muscle cross-section for satellite cell localization. A Pax7^+^ nucleus (green, *arrow*) located outside the dystrophin^+^ (red) plasma membrane of a myofibre. DAPI (blue) shows the nuclei. Scale bar = 20 µm. (C) Total Pax7 immunoreactive nuclei; BrdU^+^/Pax7^+^ double-positive nuclei per 10^3^ myofibers; and ratio of BrdU^+^/Pax7^+^ nuclei within the total Pax7^+^ population. *p<0.05, significant difference compared to WB group; ^#^p<0.05, significant difference compared to HU group. Values are means ± SEM.

To examine the changes in the total satellite cell pool as well as proliferating satellite cells upon hindlimb unloading, we measured Pax7-immunoreactive nuclei and co-labeled with BrdU incorporation (Fig. 2A). Satellite cell counting was based on Pax7^+^ nucleus located outside of the plasma membrane labeled with dystrophin (Fig. 2B). We found that the total satellite cell number, indicated by Pax7^+^ nuclei, was decreased by 36.3% ± 9.3% after 14 days of hindlimb unloading (Fig. 2C). Furthermore, the total proliferated satellite cell population revealed by BrdU^+^/Pax7^+^ double-positive nuclei was substantially reduced by 64.0% ± 12.0% upon hindlimb suspension. To investigate if there was any alteration in the proliferative capacity within the satellite cell pool, we determined the ratio of BrdU^+^/Pax7^+^ double-positive nuclei among the Pax7-immunoreactive population. Here we observed a 26.1% ± 6.0% reduction in the proportion of BrdU^+^/Pax7^+^ nuclei among the Pax7^+^ satellite cell pool counted. Electrical stimulation (HU-ES) not only prevented the decline of the satellite cell pool (Fig. 2C), but also significantly induced proliferation of satellite cells (BrdU^+^/Pax7^+^, p<0.001). Compared to the HU group, the ratio of BrdU^+^/Pax7^+^ nuclei within the total Pax7^+^ population in the HU-ES group was increased by 80.2% ± 6.8%. In fact, the ratio was higher (33.2% ± 5.0%, p<0.05) than that of WB group.

### Satellite cell apoptosis

**Figure 3:**
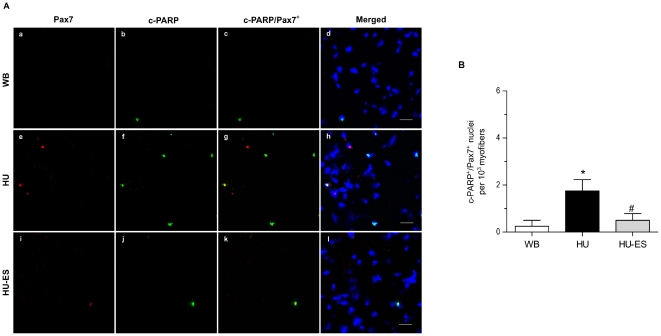
Satellite cell apoptosis. (A) Immunohistochemically stained cross-sections of soleus muscle from weight-bearing (WB), hindlimb-unloaded (HU), and electrically stimulated (HU-ES) groups showing expression of Pax7 (red) and cleaved PARP (c-PARP, green) counterstained with DAPI (blue) for nuclei identification. Yellow staining represents Pax7 and cleaved PARP double-positive nuclei. Scale bar = 25 µm. (B) c-PARP^+^/Pax7^+^ double-positive nuclei per 10^3^ myofibers for different groups. *p<0.05, significant difference compared to WB group; ^#^p<0.05, significant difference compared to HU group. Values are means ± SEM.

To determine the extent of apoptosis in the satellite cell pool induced by hindlimb unloading, we first examined the expression of the specific satellite cell marker Pax7. However, when cells are in the late stage of apoptosis, Pax7 may no longer be expressed. Therefore, we used the apoptotic marker cleaved PARP (c-PARP) and double labeled with Pax7 to detect apoptotic Pax7-expressing satellite cells (Fig. 3A). As a consequence of unloading, there was a six-fold increase (p<0.05) in the number of c-PARP^+^/Pax7^+^ nuclei (Fig. 3A-B). However, electrical stimulation (HU-ES) was able to reverse the unloading-induced apoptosis to levels similar to that in the WB group (Fig. 3B). Results from western blotting also confirmed the observations obtained from immunostaining, in which protein expression of c-PARP was significantly upregulated in the HU group but was substantially suppressed in the HU-ES group (Fig. 3C).

### TUNEL index of myonuclei

**Figure 4:**
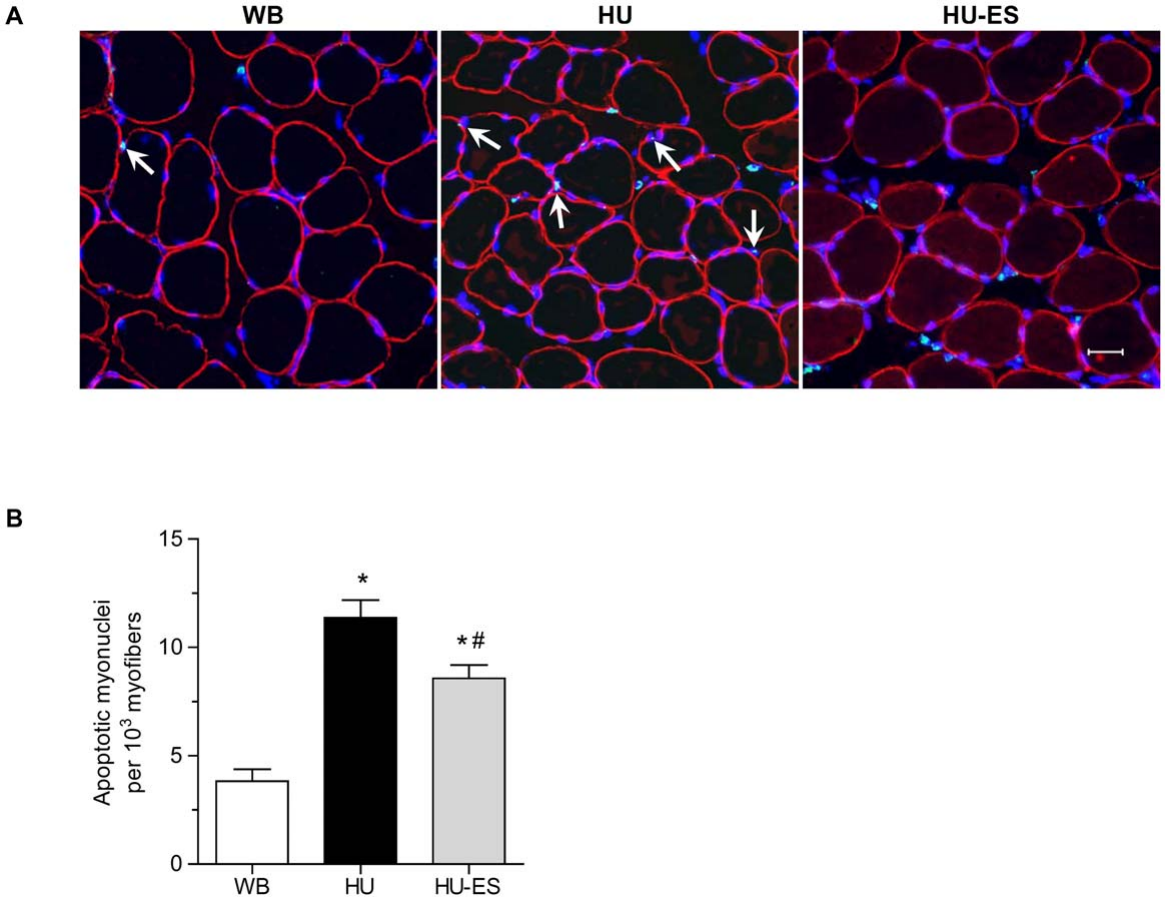
TUNEL analysis in soleus myofibers. (A) Representative images showing cross-sections of soleus muscle from weight-bearing (WB), hindlimb-unloaded (HU), and electrically stimulated (HU-ES) groups with TUNEL labeling (green, arrows) counterstained with DAPI (blue) for nuclei identification. Antibodies stained against dystrophin (red) for myofiber visualization. Scale bar = 25 µm. (B) Apoptotic index is expressed as the number of apoptotic myonuclei per 10^3^ myofibers. *p<0.05, significant difference compared to WB group; ^#^p<0.05, significant difference compared to HU group. Values are means ± SEM.

We used the TUNEL technique to identify the apoptotic myonuclei, and also labeled the sarcolemma by immunostaining against the dystrophin protein to facilitate myofiber visualization for fiber counting (Fig. 4B). The apoptotic index, represented by the total number of apoptotic myonuclei per 1000 myofibers of soleus muscles, was significantly increased (p<0.001) in the HU-group (Fig. 4A), which amounted to an increase by a factor of 1.96 ± 0.21 when compared to WB controls (Fig. 4B). Electric stimulation significantly reduced the apoptotic index in the HU-ES group by 24.6% ± 5.4% compared to the HU group, though it was still higher than WB controls (p<0.05).

### Apoptosis-related proteins

**Figure 5:**
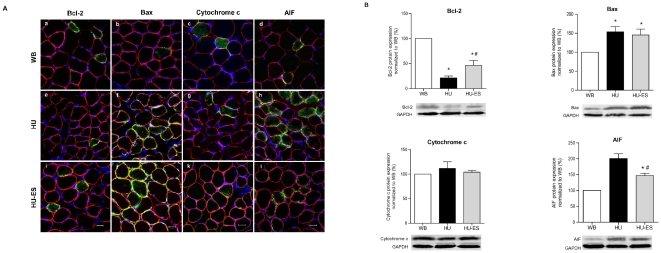
Bcl-2, Bax, cytochrome c, and AIF of the soleus muscle in the weight-bearing (WB), hindlimb-unloaded (HU), and electrically stimulated (HU-ES) groups. (A) Immunohistochemically stained cross-sections showing expression of Bcl-2, Bax, cytochrome c, and AIF (all in green) counterstained with antibodies against dystrophin (red) for myofiber identification and DAPI (blue) for nuclei visualization. Scale bar = 25 µm. (B) Western blot analysis of expression of Bcl-2, Bax, cytochrome c, and AIF relative protein levels normalized to WB group. Representative immunoreactive bands were shown. *p<0.05, significant difference compared to WB group; ^#^p<0.05, significant difference compared to HU group. Values are means ± SEM.

Bax, Bcl-2, AIF, and cytochrome c proteins play important roles in the regulation of the mitochondrial pathway of apoptosis. Changes in expression levels of these regulatory proteins reflect the impairment of the cell's survival programs. To investigate if there was any change in expression level of the above proteins in the soleus muscles after hindlimb unloading, and whether electrical stimulation was able to suppress the altered expression level, immunostaining and western blotting were performed. Myofibers immunopositive for Bcl-2, Bax, cytochrome c, and AIF were all detected in WB controls (Fig. 5A), but the expression was differentially regulated upon hindlimb unloading. In the HU group, Bax- and AIF-immunopositive myofibers were more frequently detected (p<0.001). Compatible with the immunohistochemistry findings, western blotting also showed a significant increase in the expression levels of Bax and AIF (p<0.05 for Bax; p<0.001 for AIF). When subjected to electrical stimulation, expression of AIF was reduced by 27% ± 3.8% in the HU-ES group compared to the HU group. Although no significant difference was demonstrated in pro-apoptotic (Bax) protein expression between the HU and HU-ES groups, an opposite trend was observed in anti-apoptotic (Bcl-2) protein expression in the HU group, in which Bcl-2 protein was reduced by 78.7% ± 3.8%. Electrical stimulation (HU-ES) induced a one-fold increase in Bcl-2 expression, although the level was still far from those in WB controls. No significant differences were observed in cytochrome c content between the WB, HU, and HU-ES groups (Fig. 5).

**Figure 6:**
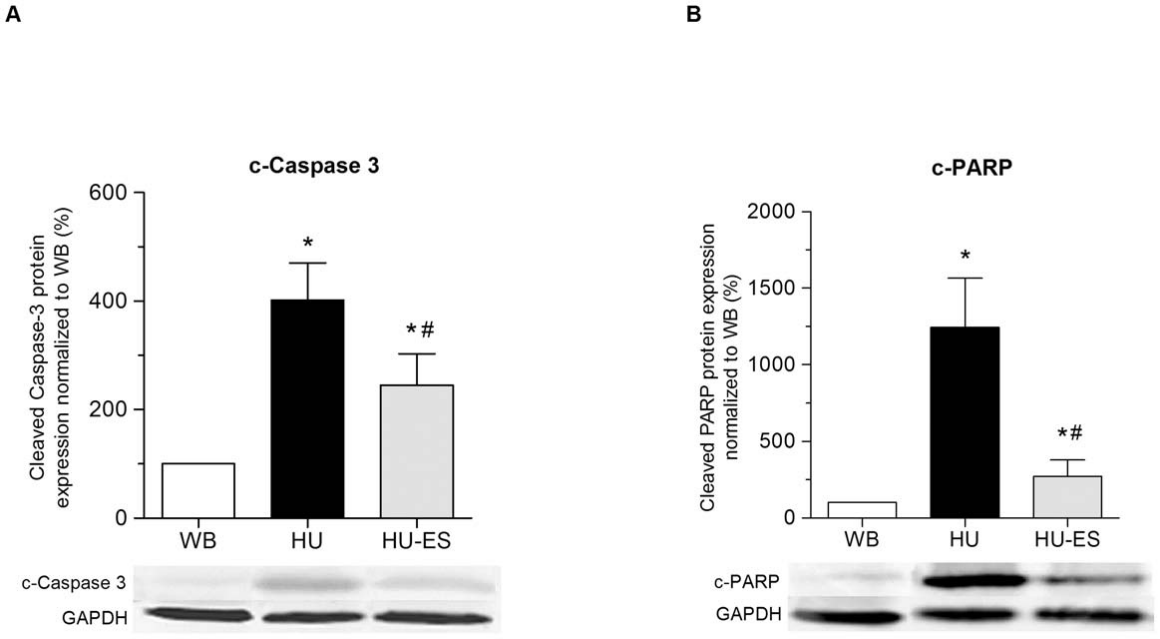
Expression levels of cleaved forms of caspase-3 and PARP in the soleus muscle obtained from weight-bearing (WB), hindlimb-unloading (HU), and electrically stimulated (HU-ES) groups. (A) Western blot analysis of expression of cleaved caspase-3. (B) Western blot analysis of expression of cleaved PARP. The relative protein levels were normalized to WB group. Representative immunoreactive bands were shown. *p<0.05, significant difference compared to WB group; ^#^p<0.05, significant difference compared to HU group. Values are means ± SEM.

To further investigate the involvement of the downstream signaling cascades of apoptosis, expression of the downstream cleavage forms of caspase-3 and PARP were examined. Upon hindlimb unloading, both cleaved caspase-3 and c-PARP were significantly upregulated in the HU group (p<0.01). Electrical stimulation resulted in reduced expression of cleaved caspase-3 and c-PARP in the HU-ES group compared to the HU group (Fig. 6), with c-PARP showing a more pronounced reduction.

## Discussion

Mechanical unloading is associated with detrimental changes to the structure and function of skeletal muscles, characterized by reduction in muscle mass, myofiber cross-sectional area, contractile strength and speed, as well as slow-to-fast fiber type transformation (for review, see [24]). It has also been reported that satellite cells in unloaded muscles exhibited an impaired regenerative capacity [7], [9]. We previously observed a reduction in the number of quiescent and mitotically active satellite cells during unloading, and electrical stimulation partially attenuated muscle atrophy by enhancing satellite cell proliferation and activation [19]. In that study, we reported a reduction in the myonuclear domain and number during unloading. Electrical stimulation partially prevented the loss in the myonuclear domain but failed to restore the myonuclear number. Regulation of the myonuclei turnover is associated with addition (satellite cell-mediated myonuclei accretion) and loss (by apoptosis) of myonuclei, depending on the muscle activity and ongoing physiological process [25]. In the present study, we sought to investigate the contribution of electrical stimulation in regulating the myonuclei turnover through improving proliferation of satellite cells and inhibiting cell apoptosis during the muscle atrophic process.

In this study, electrical stimulation resulted in a significant improvement in soleus muscle mass and cross-sectional area compared to that observed in the unloaded muscles, and restored muscle mass to approximately 80% of the WB controls. This is similar to our previous observation [19]. To further characterize the processes that might be associated with the observed electrical stimulation-induced changes, we evaluated the contractile properties of the soleus muscle by measuring changes in: (i) P_o_; (ii) length-tension relation; and (iii) force-frequency relation. Following unloading, we observed a reduction in P_o_, and the peak length-tension relation was generated at a shorter muscle length. This phenomenon has been shown in disuse-related atrophy as a consequence of loss in sarcomere number in series [26], [27], [28]. Furthermore, we observed a greater relative reduction in force at low frequencies of stimulation compared to high. This phenomenon, first described by Edwards and colleagues [29], is known as low-frequency fatigue and is suggested to be caused by a defect in excitation-contraction coupling. We found that this paradigm of electrical stimulation produced some improvement in the mechanical properties of the muscles.

It has been reported that hindlimb suspension resulted in myonuclei undergoing apoptosis [30]. We examined the extent of apoptosis in myonuclei and found a substantial increase in the number of apoptotic myonuclei. According to the myonuclei domain hypothesis, such a dramatic increase in apoptotic myonuclei should accompany an increase in muscle fibers exhibiting pro-apoptotic features. Apoptosis can be triggered through two major pathways: the death receptor pathway, which involves Fas, TNF-related apoptosis-inducing ligand, and TNF receptors; or the mitochondrial pathway, where Bax and Bcl-2 are the center of focus [31]. Since limited evidence has been demonstrated regarding the involvement of the death receptor pathway on eliciting apoptosis in atrophic muscles, focus was given to the mitochondrial pathway. Indeed, we observed a significant increase in pro-apoptotic Bax and AIF protein expression, as well as in the effector proteins cleaved caspase-3 and c-PARP, in unloaded myofibers. It is suggested that during the onset of apoptosis, Bax-Bax oligomerization takes place and the homomers translocate into the outer mitochondrial membrane, and through modulating the mitochondrial membrane permeabilization, mediates the release of cytochrome c (caspase dependent) and AIF (caspase independent) from the mitochondrial intermembrane space into the cells via the transitional pores [5], [32]. Depending on the involvement of caspase, cytochrome c and AIF separately trigger the subsequent signaling molecules and eventually lead to DNA fragmentation. The process of apoptosis can be further fine-tuned, in which Bax-Bax oligomerization can be counteracted by an anti-apoptotic Bcl-2 protein, thereby blocking the formation of transitional pores and thus inhibiting apoptosis [33], [34], [35]. The interactions between Bax and Bcl-2, therefore, determine the onset of apoptosis. As expected, a corresponding decrease in anti-apoptotic Bcl-2 protein expression was also detected in myofibers upon unloading. The differential expression of Bax and Bcl-2 protein clearly indicated that the mitochondrial-specific apoptotic pathway was activated by unloading. Electrical stimulation rescued the myonuclei from undergoing apoptosis, apparently by enhancing the Bcl-2 protein expression that suppressed Bax oligomerization without altering the expression of Bax, thus accounting for the partial recovery of the myonuclei domain.

Since satellite cells are the predominant source of muscle regeneration, it seems that the loss of myonuclei would be replenished by the activation and myogenic differentiation of satellite cells. We mapped the quiescent and activated satellite cells using Pax7, a specific marker expressed only in quiescent and proliferating satellite cells but absent in newly regenerated myonuclei [36], [37], [38]. In addition to the existing satellite cells exhibiting defective myogenesis as previously shown upon unloading [19], we observed that satellite cells also committed into apoptosis, as indicated by the increase in satellite cell population expressing c-PARP. The cleavage of PARP is suggested to be an early event in apoptosis, in which the detection of PARP cleavage is earlier than other apoptosis-related events; e.g., DNA fragmentation [39]. Furthermore, the proliferative potential of satellite cells was also impaired during unloading, as seen in the diminished BrdU^+^/Pax7^+^ subpopulation in hindlimb unloaded muscles. The underlying mechanism of extensive apoptosis in satellite cells is not entirely clear. One of the possible explanations might be the alteration in the environment of satellite cells during the atrophic process. Oxidative stress plays an important role in the development of apoptosis and is implicated in muscle atrophy. An increase in lipid peroxidation was observed in mice following hindlimb unloading [40], and furthermore the level of lipid peroxidation was linearly related to the percentage of muscle atrophy [41]. It was speculated that unloading-induced lesions observed in the central core of the soleus muscle was similar to the human central core disease [42], which is linked to mutations of muscle-specific Ca^2+^-releasing channels, and resulted in excessive release of Ca^2+^ that led to Ca^2+^ overload [43]. Reactive oxygen species (ROS) accumulation has been correlated Ca^2+^ release from the sarcoplasmic reticulum and lead to activation of the Ca^2+^-dependent pathways. The increase in the activity of Ca^2+^-dependent protease (i.e. calpain) induced proteolysis in muscles [44], [45]. A recent study in human satellite cells has demonstrated that oxidative stress-induced calpain-dependent pathway resulted in the depletion of the satellite cell pool [46]. Together, increasing commitment of myonuclei and satellite cells into apoptosis as well as the deficient proliferative capacity of satellite cells may be the crucial if not sole factor that limits muscle repair, thus accounting for the atrophic features in the unloaded muscles.

Passive stretch of the unloaded soleus muscle have been shown to increase satellite cell proliferation [47] and maintenance of the satellite cell numbers [42]. It was speculated that stretch might prevent Ca^2+^ accumulation by reducing the overlap of thick and thin filaments, and consequently promote diffusion and removal of Ca^2+^ [42]. Cross talk between the mitochondrial pathway and calpain has been documented, in which calpain promoted apoptosis by inducing cleavage of the Bcl-2 protein [48]. Both stretching and electrical stimulation worked similarly by generating tension required by the slow-twitch muscle, an explanation of our finding that electrical stimulation improved muscle function might be the suppression of satellite cells apoptosis through inhibition of Ca^2+^ accumulation and hence blocked the calpain-mediated Bcl-2 cleavage. The effect of electrical stimulation on the regulation of calpain activity needs further investigation.

Although there was a significant improvement of electrical stimulation in attenuating the atrophic features upon unloading, the functional and morphological parameters did not reach normal levels. Given that numerous mechanisms and complex interplay of signal transduction pathways are involved in regulating muscle atrophy [49], it is likely that a combined (rather than a single) intervention approach should be considered. Interestingly, It has been demonstrated that satellite cell proliferation increased dramatically in the first 6 hours after unloading followed by a slight decline at 12 hours, the proportion of proliferating satellite cells was comparable to control by 48 hours after unloading. From that onwards, the population of proliferating satellite cells decreased far below that of the control group [11]. It was believed that a sudden loss of microgravity initially triggered a signal to initiate satellite activation and proliferation ready for subsequent muscle repair. However, such proliferation could not be sustained without appropriate mechanical stimuli. In the present study, the tension generated by electrical stimulation, together with the stimulation frequency which matched the motor unit firing pattern of slow-twitch soleus muscle, provided the required mechanical signal to promote satellite cell proliferation and protect the cells from undergoing apoptosis. It is possible that the favorable effect of electrical stimulation on satellite cell proliferation, as evidenced by the recovery of the entire Pax7-immunopositive satellite cell pool, secures a healthy and sufficient pool of reserve (i.e. satellite cells) for subsequent muscle regeneration when appropriate mechanical stimuli is provided (i.e. reloading).

In conclusion, the present study describes the effect of electrical stimulation on maintaining the satellite cell pool through regulation of cell proliferation and apoptosis during hindlimb unloading-induced atrophy. Electrical stimulation may prove a useful adjunct intervention in controlling skeletal muscle mass. Further optimization of the stimulation parameters may help to improve the regenerative capacity of the muscles.
